# Supplementary material for: Content-rich biological network constructed by mining PubMed abstracts
Source: BMC Bioinformatics. 2004 Oct 8;5:147. doi: 10.1186/1471-2105-5-147 (PMC528731; doi:10.1186/1471-2105-5-147)
Supplement: Additional File 5 — The original Chilibot query results of the term "long-term potentiation (LTP)" and 22 other terms, limiting the latest references analyzed to the years 1990, 1995, 2000, and 2004. [file 1471-2105-5-147-S5.bz2 › chilibotAdditionalFile5/ltp2000/html/PI-3K.html]

 


**PI-3K** (Input: PI-3K ) 

---


**Google Searches:** Entire Web  | EDU domain only  | PDF files only

.

**External Links:** OMIM |
LocusLink
| Swissprot
| GeneCards

  
**Maps of PI-3K**

Simple
Complete
graph in
radiant
tree
square
layout.

**New Hypothesis !**

**Synonyms** 

- pi-3k   [PubMed]

- pi-3-kinase   [PubMed]

- pi3-kinase   [PubMed]

**Synopsis**

- These results suggest that the PI 3 kinase  [**PI-3K**]  pathway plays an important role in signaling by collagen and in the priming action of TPO.  Blood, 2000 
  [23]

- the obtained data suggest that an activation of alpha1A adrenoceptors, presence in WAT, by agonist and or neurotransmitter may increase the glucose uptake via PLC PKC pathway and the activation of PI 3 kinase  [**PI-3K**] .  Auton Neurosci, 2000 
  [20]

- Taken together, our results suggest that the peptide activate ERK via a G protein PI 3K  [**PI-3K**]  Ras Raf 1 mediated signaling pathway in U937 cells.  Life Sci, 1999 
  [20]

- These results suggest that in primary endothelial cells, VEGF induced activation of Raf MEK MAP kinase and DNA synthesis are mainly mediated by PKC dependent pathway, much more than by Ras dependent or PI3 kinase  [**PI-3K**]  dependent pathway.  Oncogene, 1999 
  [20]

- incontrast, PLC inhibitor U 73122 did suppress adhesion, suggesting the possibility that PLC,   Cell Immunol, 1998 
  [19]

- The evidence presented is consistent with the hypothesis that PI 3 kinase  [**PI-3K**]  activation, possibly by NGF, plays a role in expression of LTP in dentate gyrus.  Neuropharmacology, 2000 
  [16]

- The nascent models highlight the roles of various intracellular signaling pathways including phospholipase C gamma PLC gamma, phosphatidylinositol PI 3 kinase  [**PI-3K**] , mitogen activated protein MAP kinase, and actin cytoskeleton related events.  Adv Cancer Res, 2000 
  [16]

- Moreover, this study bears out the notion that individual PI 3 kinase  [**PI-3K**]  lipid products play distinct roles in the regulation of cellular functions.  Biochemistry, 1998 
  [16]

- We propose that PI 3 kinase  [**PI-3K**]  and its product PtdIns,5 P3 play a key role in the activation and adequate location of PLCgamma2 induced by FcgammaRIIA cross linking.  J Biol Chem, 1998 
  [16]

- In addition, villin was found to associate with the 85 KDa subunit p85 of phosphatidylinositol PI 3 kinase  [**PI-3K**] , concomitant with PLC gammal, in the cytoskeletal fraction of Na P i cotransport up regulated cells.  Mol Med, 2000 
  [15]

- Using immunoprecipitation, PI3 kinase  [**PI-3K**]  and tyrosine phosphorylated PLC gamma1 and SHC were shown to be associated with tyrosine phosphorylated TrkB in response to both BDNF and NT 4.  Exp Neurol, 1999 
  [15]

- These results demonstrate that PI 3 kinase  [**PI-3K**]  is required for full activation of PLCgamma2 by GPVI in platelets and megakaryocytes.  Biochem J, 1999 
  [14]

- Thus, NPM ALK activates the antiapoptotic PI 3 kinase  [**PI-3K**]  Akt pathway, which likely contributes to the molecular pathogenesis of ALCL.  Blood, 2000 
  [10]

- incontrast to cytokine primed cells, only the tyrosine kinase activated isoform of PI3 kinase  [**PI-3K**]  is coupled to FcgammaRI in dbcAMP differentiated cells.  Immunology, 1999 
  [10]

- The HGF receptor directly activates PI3 kinase  [**PI-3K**] , Ras and STAT signalling pathways and phosphorylates the adaptator GRB2 Associated Binder 1 Gab1 .  Oncogene, 2000 
  [10]
